# Retrospective analysis of tacrolimus combined with *Tripterygium wilfordii* polyglycoside for treating idiopathic membranous nephropathy

**DOI:** 10.1186/s12882-018-0967-5

**Published:** 2018-07-18

**Authors:** Shun-Lai Shang, Guang-Yan Cai, Shu-wei Duan, Ping Li, Qing-Gang Li, Xiang-Mei Chen

**Affiliations:** Department of Nephrology, Medical School of Chinese PLA, Chinese PLA Institute of Nephrology, State Key Laboratory of Kidney Diseases, National Clinical Research Center for Kidney Diseases, Chinese PLA General Hospital, 28 Fuxing Road, Haidian District, Beijing, 100853 China

**Keywords:** Tacrolimus, *Tripterygium wilfordii* polyglycoside, Idiopathic membranous nephropathy, Methylprednisolone

## Abstract

**Background:**

Idiopathic membranous nephropathy (IMN) is one of the most common adult nephrotic syndromes. Some patients with this disorder require immunosuppressive therapy. This retrospective case series was performed to assess the effects of tacrolimus (TAC) combined with *Tripterygium wilfordii* polyglycoside (TWG) in treating IMN.

**Methods:**

From January 2015 to August 2016, kidney-biopsy-proven IMN patients treated with TAC in the Chinese PLA General Hospital were screened. Data were retrieved from the patients’ medical records. The first efficacy evaluation index was remission rate (complete remission and partial remission), and the secondary efficacy evaluation indices included relapse rate, proteinuria, serum albumin and estimated glomerular filtration rate (eGFR). Adverse events were also assessed.

**Results:**

The included patients’ treatments were tacrolimus monotherapy (TAC group, *n* = 33), tacrolimus combined with methylprednisolone (MP) (TAC + MP group, *n* = 24) and tacrolimus combined with *Tripterygium wilfordii* polyglycoside (TAC + TWG group, *n* = 21). The remission rates of the TAC, TAC + MP, and TAC + TWG groups in the 10th month were 54.5, 62.5, and 85.7%, respectively (TAC + TWG group vs TAC group, *P* = 0.037, TAC + TWG group vs TAC + MP group, *P* = 0.125). Moreover, the complete remission rates of the TAC, TAC + MP, and TAC + TWG groups in the 10th month were 21.2, 20.8, and 57.1%, respectively (TAC + TWG group vs TAC group, *P* = 0.007, TAC + TWG group vs TAC + MP group, *P* = 0.012). Compared with the TAC group, the TAC + TWG group had a higher remission rate during these ten months (log-rank, *P* = 0.005). Compared with the TAC and TAC + MP groups, the TAC + TWG group had a higher complete remission rate (log-rank, *P* = 0.019 and log-rank, *P* = 0.005, respectively).

**Conclusion:**

This retrospective study showed that TAC combined with TWG may be effective for treating IMN. Further randomized controlled trials (RCTs) are needed to assess the efficacy and safety of TAC combined with TWG.

## Background

Approximately 34–62% of idiopathic membranous nephropathy (IMN) cases will progress to renal insufficiency [1]. Immunosuppressive therapies, such as cyclosporine A, tacrolimus (TAC), and mycophenolate mofetil, have been shown to induce remission and reduce the risk of progression to end-stage renal disease or death [2, 3]. However, use of these immunosuppressants is controversial [4] due to their side effects, which can be detrimental and include bone marrow suppression, infection, and thrombosis formation [5]. In 2007, results from a randomized controlled trial (RCT) showed that tacrolimus effectively treated IMN [6]; however, the tacrolimus was expensive and caused nephrotoxicity and high relapse rates after being discontinued [7]. In 2016, another RCT found that the efficacy of tacrolimus combined with glucocorticoids was comparable to that of cyclophosphamide combined with glucocorticoids, but the former therapy resulted in more adverse effects, such as nephrotoxicity and corticosteroid-related side effects [8].

*Tripterygium wilfordii* polyglycoside (TWG) is an extract from the traditional Chinese medicinal plant, *Tripterygium wilfordii* [9], which has anti-inflammatory and immunosuppressive effects [10, 11]. Studies have shown that TWG can inhibit NO production and iNOS expression by blocking NF-κB activation. Additionally, TWG can inhibit lymphocyte proliferation [12]. Other studies have shown that TWG regulates inflammatory reactions mediated by Toll-like receptors (TLR) and decreases dangerous chronic diseases that are correlated with exaggerated TLR activation [13]. Other studies demonstrated that TWG modulated triggering receptors expressed in the myeloid cell (TREM)-1 signaling pathway to inhibit the inflammatory response in rheumatoid arthritis (RA), yielding a good effect [14, 15]. TWG treatment can also reduce inflammatory cytokine expression in serum and the kidneys and relieve nephropathy in diabetic rats [16]. Recently, Liu S et al. found that TWG combined with corticosteroid therapy was a more effective strategy for IMN [17]; however, it still produced corticosteroid-related side effects. Therefore, we performed this retrospective study to investigate the effects of TAC combined with TWG on IMN, as these two drugs are based on different treatment mechanisms.

## Methods

### Study population and oversight

This study was a retrospective study conducted from January 1, 2015 to August 1, 2016, in which all biopsy-proven IMN cases treated with tacrolimus were screened at Chinese PLA General Hospital. The other criteria for collecting patients’ data were as follows: (1) aged 18 to 70 years old of either gender; (2) treated without glucocorticoid or immunosuppressive therapy for at least 5 months and a urinary protein level >3.5 g; and (3) an eGFR Chronic Kidney Disease Epidemiology Collaboration (CKD-EPI) greater than 50 ml/min/1.73 m^2^. We excluded patients with histories of autoimmune diseases, cancer and secondary membranous nephropathies, such as systemic lupus erythematosus and hepatitis B virus (HBV)-associated nephropathy. The study was approved by the Chinese PLA General Hospital Medical Ethics Committee. Signed informed consents were obtained from all the patients.

### Data collection

We collected data on demographics, laboratory test results, renal pathology, side effects and follow-up from the patients’ medical records. The follow-up time was 10 months. The monthly laboratory tests included serum creatinine, eGFR, serum albumin, alanine aminotransferase, urinary protein quantification, systolic blood pressure, diastolic blood pressure, antibody against m-type phospholipase A2 receptor (anti-PLA2R) and the tacrolimus blood concentration. Serum anti-PLA2R levels were measured using previously published and validated methods produced by EUROIMMUN AG (Germany), an enzyme-linked immunosorbent assay (ELISA) performed per the manufacturer’s instructions. The serum anti-PLA2R antibody range was from 2 to 1500 RU/mL, and a positive result was concluded when the concentration was > 20 RU/mL. Renal pathological data included IMN stage, semi-quantitative score [18] and immunofluorescence deposit score [19].

### Treatment method

Patients in the TAC group initially received TAC at a dosage of 0.045 to 0.06 mg/kg per day, and the TAC was divided into two equal doses taken once every 12 h. The tacrolimus blood concentration was detected routinely at 15 days. The pre-dose trough concentration was routinely monitored during treatment. The TAC doses were adjusted by blood concentration, with a target range of 4–8 ng/ml for at least 6 months, which was then reduced gradually. The tacrolimus dosage reduction was decided by clinicians based on its therapeutic effect and the patients’ tolerance. If the targeted tacrolimus concentration level was not achieved, the dosage was adjusted, and the concentration was detected at the next follow-up. For patients in the TAC + MP group, the dose and use of TAC were the same as for the TAC group. Patients also initially received oral methylprednisolone (MP) at 0.5 mg/kg per day for the first 2 months. The dose was then reduced by 5 mg per half month until reaching a dose of 20 mg, which was maintained for 2 months, and then the dose was reduced again slowly over 3 months until withdraw. The TAC dose and use in the TAC + TWG group were the same as for the above two groups. These patients were additionally administered 20 mg of TWG orally 3 times per day for at least 6 months [17]. The TWG was then reduced gradually as determined by clinicians based on the treatment effects and patient tolerance.

### Efficacy evaluation and endpoint events

(1) Complete remission was urinary protein less than 0.3 g/d at least twice per week, with normal serum albumin and normal serum creatinine (SCr). (2) Partial remission was urinary protein less than 3.5 g/d and at least 50% lower than the peak, with normal or improved serum albumin and stable SCr. (3) Patients who did not meet the above conditions were considered invalid. (4) Relapse was a urinary protein level greater than 3.5 g/d and more than 50% higher than the lowest urinary protein level after remission. (5) Renal failure was when serum creatinine was raised by more than 50% above the baseline.

### Statistical analysis

(1) Continuous variables with normal distributions were expressed as the mean ± SD. Comparisons between two groups were conducted using a t-test. Comparisons among multiple groups were conducted using a one-way analysis of variance (ANOVA). (2) Continuous variables with discrete distributions were expressed as the median (M), quartile spacing (Qu-Ql) and the mean ± SD and were tested using a nonparametric rank-sum test. (3) Categorical variables were expressed as absolute values (percentages). Comparisons between groups were conducted using the Pearson χ^2^ test or Fisher’s exact test. The TAC + TWG group remission rate was compared with those of the TAC and TAC + MP groups. (4). Survival data were analyzed by the log-rank test. All data were analyzed using SPSS 23.0 software.

## Results

**Fig. 1:**
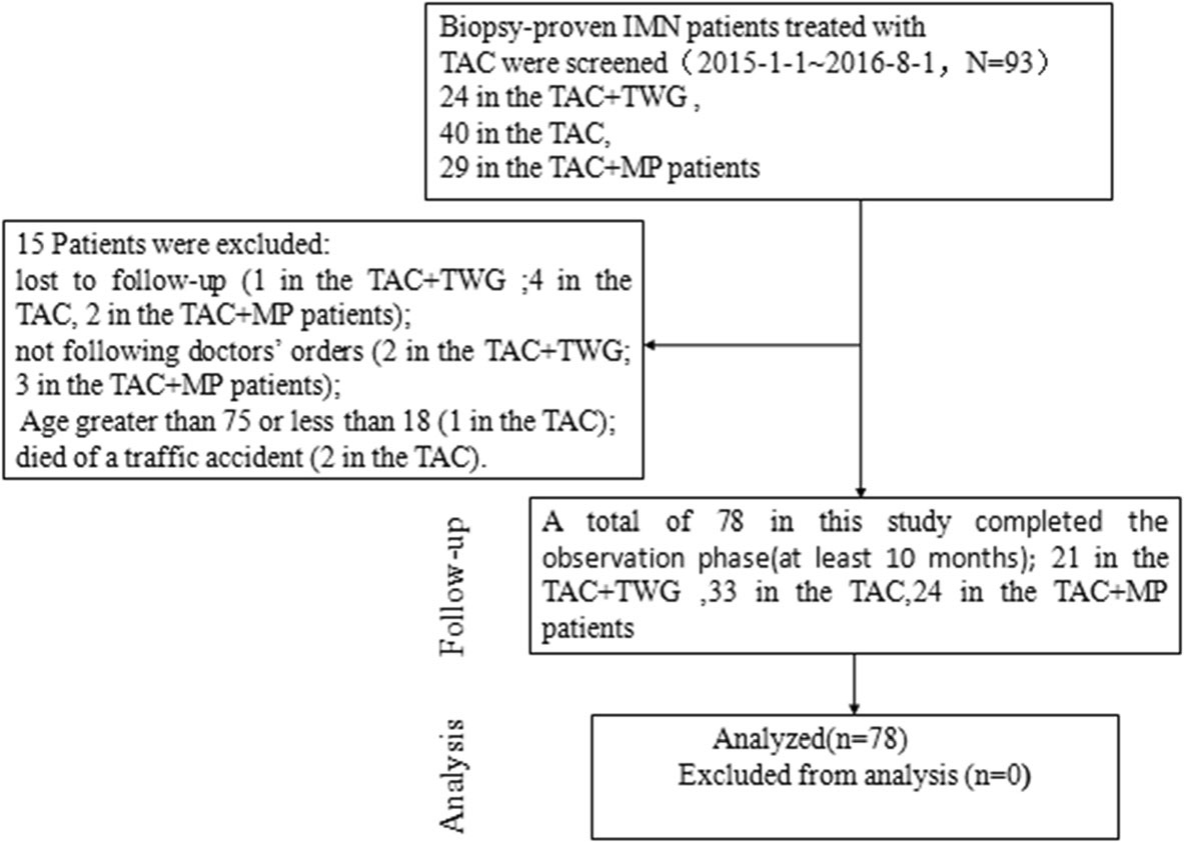
Trial flow

From January 2015 to August 2016, 93 biopsy-proven IMN cases were treated with TAC at Chinese PLA General Hospital, including 40 patients who received TAC alone, 24 who received TAC + TMG, and 29 who received TAC + MP. Of these, 78 were enrolled in the study and completed the observation phase (minimum 10 months). Reasons for exclusion included being aged over 75 years (1 in the TAC group); lost to follow-up (1 in the TAC + TWG, 4 in the TAC, and 2 in the TAC + MP groups); not following the doctors’ prescriptions (2 in the TAC + TWG and 3 in the TAC + MP groups); and died in a traffic accident (2 in the TAC group). The screening flowchart is shown in Fig.1.

### Baseline characteristics

The clinical and pathological baseline characteristics of the three groups are shown in Table 1.

**Table 1:** Baseline characteristics of the patients in the three groups

Characteristics	TAC group (*n* = 33)	TAC + MP group (*n* = 24)	TAC + TWG group (*n* = 21)	*P* value
Sex (male/female)	13/20	14/10	9/12	0.35
Age (years)	40(21)	40(26)	50(22)	0.16
	42.00 ± 15.25	43.29 ± 14.59	49.43 ± 11.89	
BMI (kg/m^2^)	25.30(4.85)	24.85(5.82)	23.70(5.10)	0.37
	25.82 ± 5.38	25.91 ± 3.59	24.85 ± 5.05	
Systolic BP (mmHg)	129.15 ± 15.38	131.00 ± 15.65	131.00 ± 20.48	0.93
Diastolic BP (mmHg)	71.73 ± 16.77	71.83 ± 16.14	69.29 ± 15.51	0.62
Daily urinary protein (g/24 h)	6.07(2.43)	7.38(2.87)	5.92(2.19)	0.387
	6.95 ± 2.43	7.25 ± 1.61	6.61 ± 1.34	
Serum albumin (g/L)	26.13 ± 3.31	26.07 ± 2.81	25.10 ± 3.55	0.64
Concentrations of anti-PLA2R antibodies (RU/mL)	571.00 (215.00)	571.5(61.25)	641.69(98.00)	0.018*
Anti-PLA2R (positive/negative)	32/1	23/1	20/1	1
Scr (μmol/L)	72.38 ± 19.78	77.28 ± 23.82	69.19 ± 15.53	0.57
BUN (mmol/L)	5.37 ± 1.97	6.46 ± 2.88	6.00 ± 1.78	0.16
UA (μmol/L)	358.28 ± 128.60	326.81 ± 97.13	353.62 ± 110.70	0.47
eGFR (ml/min/1.73 m^2^)	97.58 ± 25.51	97.44 ± 24.48	98.40 ± 15.85	0.99
Hb (g/L)	128.79 ± 16.42	129.67 ± 17.37	131.33 ± 22.04	0.89
PLT (10^^9^/L)	252.58 ± 63.37	257.88 ± 60.62	233.86 ± 57.8	0.39
WBC (10^^9^/L)	7.28 ± 1.74	6.97 ± 1.60	6.70 ± 1.86	0.49
Tch (mmol/L)	5.67 ± 1.95	6.13 ± 2.65	5.56 ± 0.89	0.62
TG (mmol/L)	1.77 ± 2.04	2.41 ± 1.76	2.08 ± 0.85	0.26
LDL (mmol/L)	3.57 ± 1.19	4.46 ± 2.02	3.59 ± 1.02	0.32
HDL (mmol/L)	1.50 ± 0.60	1.55 ± 0.56	1.55 ± 0.73	0.97
Blood glucose (mg/dL)	5.10 ± 0.61	4.9 ± 0.84	6.08 ± 2.38	0.37
Conservative treatment time (months) **#**	5.00(4.50)	8.00(5.00)	9.00(13.00)	0.06
	6.79 ± 2.25	8.54 ± 2.70	9.71 ± 5 .60	
ALT (U/L)	31.33 ± 5.12	33.78 ± 8.01	31.82 ± 5.27	0.33
AST (U/L)	15.72 ± 4.47	17.94 ± 10.75	18.13 ± 4.70	0.16
Glomerular stage at renal biopsy (I/I-II/II/II-III/III)	16/4/8/5	11/8/4/1	9/4/7/1	0.75
interstitial fibrosis and tubular atrophy (score)	1.00(1.00)	1.00(1.00)	1.00(1.00)	0.77
	0.85 ± 0.87	0.67 ± 0.48	0.62 ± 0,50	
Vascular sclerosis (score)	1.00(2.00)	1.00 (2.00)	0.00(2.00)	0.68
	0.91 ± 0,95	1.00 ± 1, 02	0.76 ± 1.14	
Immunohistological staining				
IgG (score)	2.00(1.50)	2.00(0.75)	2.00(2.00)	0.86
	1.49 ± 0.87	1.63 ± 0.71	1.43 ± 0.93	
IgG1 (score)	0.00(0.00)	0.00(1.00)	0.00(1.00)	0.41
	0.21 ± 0.55	0.33 ± 0.57	0.38 ± 0.67	
IgG4 (score)	0.00(0.00)	0.00(1.00)	0.00(1.00)	0.43
	0.24 ± 0.61	.0.38 ± 0.65	0.43 ± 0.75	
C3 (score)	1.00(1.00)	1.00(1.00)	0.00(1.00)	0.09
	0.73 ± 0.76	0.71 ± 0.46	0.38 ± 0.59	
C4 (score)	0.00(0.00)	0.00(0.00)	0.00(0.00)	0.83
	0.13 ± 0.42	0.08 ± 0.28	0.48 ± 0.22	
C1q (score)	0.00(0.00)	0.00(0.00)	0.00(0.00)	0.31
	0.09 ± 0.30	0.13 ± 0.45	0.19 ± 0.51	
Fib (score)	0.00(0.00)	0.00(0.00)	0.00(0.00)	0.86
	0.06 ± 0.24	0.08 ± 0.28	0.05 ± 0.22	
lgM (score)	0.00(0.00)	0.00(0.00)	0.00(0.00)	0.32
	0.03 ± 0.17	0.08 ± 0.36	0.14 ± 0.36	
IgA (score)	0.00(0.00)	0.00(0.00)	0.00(0.00)	0.99
	0.12 ± 0.42	0.08 ± 0.26	0.10 ± 0.30	

### Response to therapy

**Fig. 2:**
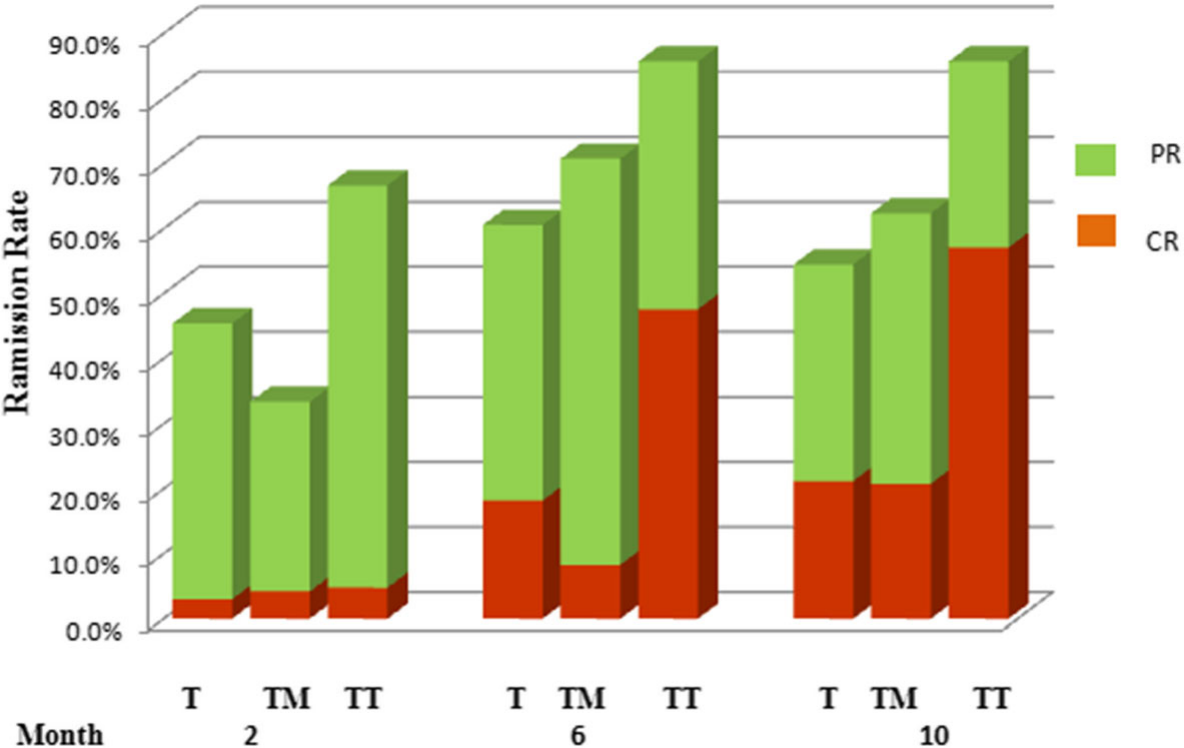
Percentage of complete (red) and partial (green) remissions in the three groups.CR, complete remission; PR, partial remission; T, TAC group; TM, TAC + MP group; TT, TAC + TWG group;

**Fig. 3:**
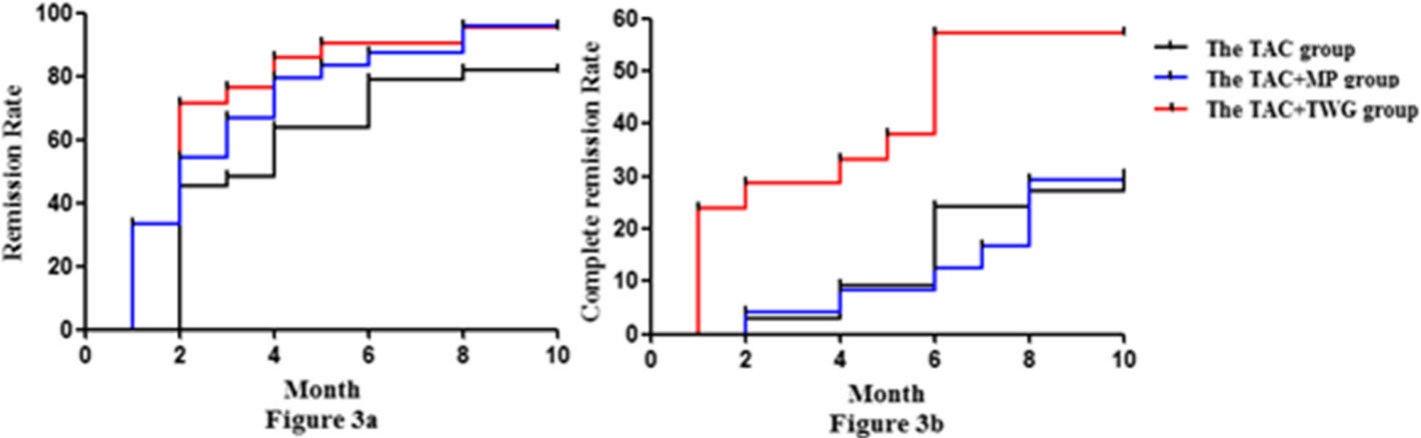
Kaplan–Meier curves of remission (partial remission and complete remission)

Compared with the TAC and TAC + MP groups, the TAC + TWG group had higher complete remission rates after 6 and 10 months, and the remission rates in the TAC + TWG group in 6th and 10th months were significantly higher than those of the TAC group. The remission results (complete and partial remission) and complete remission in the three groups during the 10 months of observation are shown in Fig. 2 and Table 2. Comparisons between the three groups and the *P* value are shown in Table 2. Compared with the TAC and TAC + MP groups, the TAC + TWG group had a higher remission rate (log-rank, *P* = 0.005 and log-rank, *P* = 0.618, respectively). In addition, the TAC + TWG group also had a higher complete remission rate than the TAC and TAC + MP groups (log-rank, *P* = 0.019, log-rank, *P* = 0.005, respectively). Time to remission and complete remission rates for the three groups are shown in Fig. 3.

**Table 2:** *P* value and remission rates of the TAC + TWG group compared with the TAC and TAC + MP groups

Project	Month	TAC group (n = 33)	TAC + MP group (n = 24)	TAC + TWG group (n = 21)	*P* ^#^	*P* ^%^
CR + PR	2th	45.4%	33.5%	66.7%	0.128	0.026
	6th	60.6%	70.5%	85.7%	0.049	0.402
	10th	54.5%	62.4%	85.7%	0.037	0.125
CR	2th	3.0%	4.2%	4.8%	0.74	0.94
	6th	18.2%	8.3%	47.6%	0.021	0.003
	10th	21.2%	20.8%	57.1%	0.007	0.012

### Secondary efficacy evaluation

#### Urinary protein

**Fig. 4:**
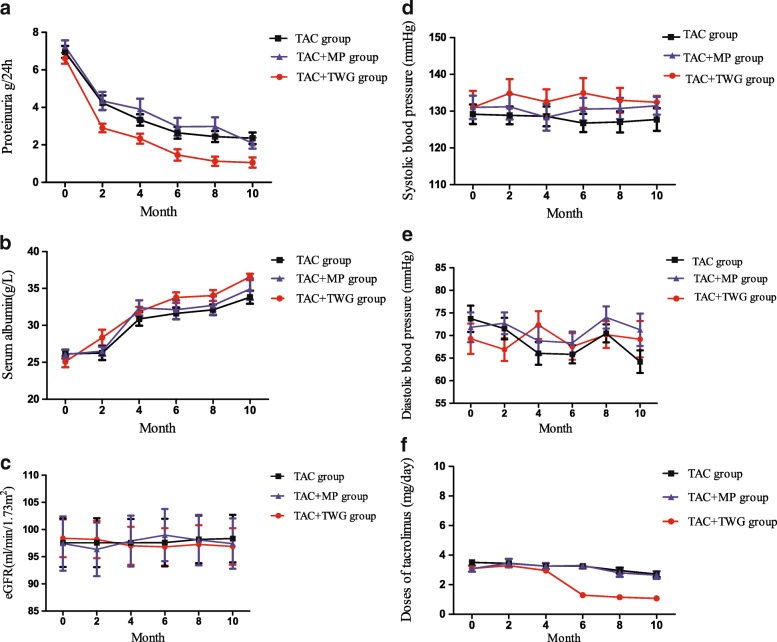
Proteinuria levels, serum albumin levels, eGFR levels, blood pressure and the TAC doses in three groups. **a** shows that the decrease in proteinuria was significantly greater in the TAC + TMG group compared with the other two groups in the 6th, 8th, and 10th months **b** shows that serum albumin increased in all three groups after treatment (*P* < 0.001). **c** to **e** respectively showed that there were no significant differences in eGFR, systolic blood pressure, and diastolic blood pressure among the three groups after treatment (*P* > 0.05). The TAC doses of the three groups are shown in (**f**)

Urinary protein decreased in all three groups after treatment (*P* < 0.05). The decrease in proteinuria was significantly greater in the TAC + TMG group than in the other two groups in the 6th, 8th, and 10th months (Table 3, Fig. 4a). Urinary protein values of less than 0.5 g were 7 (21.21%), 5 (20.83%) and 13 (61.90%) in the TAC, TAC + MP and TAC + TWG groups, respectively, in the 10th month (TAC + TWG group vs TAC group, *P* = 0.003; TAC + TWG group vs TAC + MP group, *P* = 0.005).

**Table 3:** Proteinuria levels and anti-PLA2R in the three groups

Project	Month	TAC group (n = 33)	TAC + MP group (n = 24)	TAC + TWG group (n = 21)	*P* ^#^	*P* ^%^
Daily urinary protein (g/24 h)	6th	2.51 (2.92)	2.65 (1.48)	0.32 (2.19)	0.004	0.002
		2.90 ± 1.86	3.13 ± 2.23	1.26 ± 1.21		
	8th	2.02 (2.02)	2.66(2.23)	0.29 (1.97)	0.021	0.001
		2.24 ± 1.68	3.06 ± 2.39	1.13 ± 1.11		
	10th	2.15 (2.96)	2.61 (2.54)	0.29 (1.33)	0.003	0.001
		2.35 ± 1.75	2.35 ± 1.40	1.01 ± 1.15		
anti-PLA2R antibodies (RU/ml)	10 ^th^	187.00 (293.00)	181.00 (260.75)	15 (105.00)	0.001	0.022
		169.18 ± 134.02	153.29 ± 150.21	62.19 ± 86.47

#### Albumin

After treatment, albumin levels increased in all three groups (*P* < 0.001), with no significant difference in serum albumin level between them *(P* > 0.05) (Fig. 4b). The eGFR, systolic blood pressure, and diastolic blood pressure did not significantly differ among the three groups after treatment (*P >* 0.05) (Fig. 4c, e, f).

#### TAC doses

No significant differences at baseline were seen in the 2nd or 4th months between the TAC + TWG, TAC and TAC + MP groups (*P >* 0.05). The TAC + TWG group received lower TAC doses in the 6th, 8th, and 10th months than did the TAC and TAC + MP groups (*P* < 0.001) (Fig. 4g).

#### Relapse

Compared with the TAC and TAC + MP groups, the TAC + TWG group had lower recurrence rates. Among the patients who at least experienced partial remission (PR), 1 of 21 TWG + TAC patients (4.8%), 9 of 24 TAC patients (37.5%) and 6 of 18 TAC + MP patients (33.3%) relapsed within the treatment period (TAC + TWG group vs TAC group *P* = 0.01; TAC + TWG group vs TAC + MP group, *P* = 0.024).

#### Anti-PLA2R antibody

The anti-PLA2R (positive/negative) ratios of the TAC + TWG, TAC, and TAC + MP groups in the 10th month were 4/17, 17/16, and 13/11, respectively (TAC + TWG group vs TAC group, *P* = 0.017; TAC + TWG group vs TAC + MP group, *P* = 0.015). The anti-PLA2R antibody concentrations were significantly decreased in the TAC + TMG group compared with the other two groups in the 10th month (Table 3).

### Adverse events

As shown in Tables 4, 19 adverse events occurred among the 78 patients, with 6 in the TAC + TWG group, 3 in the TAC group, and 10 in the TAC + MP group. All adverse events were mild and controllable.

**Table 4:** Adverse events among the three groups

Adverse event	TAC + TWG group	TAC group	TAC + MP group
Anemia	1	1	0
Newly developed Abnormal glucose tolerance	1	1	3
Abnormal liver function	2	1	1
Infection	1	0	2
Hypertension	0	0	0
Gastrointestinal symptoms	0	0	1
Eye disease	0	0	1
Osteoporosis	0	0	2
Bone marrow suppression	0	0	0
Irregular menstruation	1	0	0

## Discussion

Membranous nephropathy is the most common adult nephritic syndrome [20]. The most accepted pathogenesis of IMN is as follows. The immune complex activates the complement, forming the membrane attack complex, C5b-9, and stimulating inflammatory cell release in the podocytes. At the same time, C5b-9 causes glomerular hemodynamic abnormalities that affect the structural stability of the protein cytoskeleton [21, 22]. C5b-9 in the urine is inserted into the brush of the proximal convoluted tubules, causing inflammation. Finally, TGF-β and other factors promote destruction of the basement membrane integrity, causing proteinuria.

TAC mainly binds to the specific intracellular receptor, FKBP12 (FK506-binding protein 12), to form the FK506-FKBP12 complex, thereby inhibiting IL-2 [23]. TAC can also increase intracellular calcium retention and bind to the transient receptor potential cation channel 6 (TRPC6), which damages the glomerular podocytes, thereby inhibiting TRPC6 activity and reducing TRPC6-mediated damage to the glomerular podocytes [24, 25]. Tacrolimus also changes glomerular hemodynamics and decreases urinary protein [26].

Triptolide is the most important TWG constituent. TWG reduces proteinuria in experimental membranous nephropathy and protects against C5b-9-induced podocyte injury in vitro [27]. TWG alleviates glomerulosclerosis by exerting antimicroinflammatory effects, including reducing macrophage infiltration in the glomeruli, suppressing TNF-α, IL-1β and TGF-β1 overexpression in the kidney and inhibiting p38 MAPK and NF-κB signaling activities [28, 29]. Triptolide’s mechanism differs from that of prednisone and TAC [30, 31]; therefore, combining TAC and TWG is complementary, which in theory explains the combination therapy and higher remission rate in the TAC + TWG group.

In our study, the remission rates were higher in the TAC + TWG group than in the TAC group after 6 and 10 months, when the complete remission rate was significantly higher in the TAC + TWG group than in the other groups. In our study, the complete IMN remission rate was 57% in patients who received TAC combined with TWG therapy in the 10th month, which was statistically higher than the 28% observed in patients administered TAC combined with corticosteroids in a 2010 RCT [32, 33]. The Kidney Disease: Improving Global Outcomes (KDIGO) clinical practice guidelines note that half of patients on TAC treatment experience IMN relapse. In this study, the relapse rate was highest in the TAC group, thus confirming these guidelines. Conversely, the TAC + TWG group exhibited the lowest relapse rate among these treatments.

In 2016, Kohli HS et al. conducted an RCT [8], which found that the side effect reported by the TAC group was nephrotoxicity, and the eGFR level was significantly lower in the TAC group than in the CTX group. In this study, the TAC doses in the TAC + TWG group in the 6th, 8th, and 10th months were significantly lower than those in the other two groups. Further follow-up may confirm that the TAC and TAC + MP groups have a greater risk of renal insufficiency.

The M-type phospholipase A2 receptor (PLA2R) is thought to be a biomarker for serologically diagnosing IMN [34], and anti-PLA2R is important for assessing IMN disease severity. The baseline serum anti-PLA2R level is suggested to be an independent risk factor for refractory urinary protein [35]. Hoxha E et al. found that urinary protein often undergoes remission in anti-PLA2R-negative IMN [36]. Compared with the anti-PLA2R (positive/negative) and the correlation between anti-PLA2R antibody levels in the TAC and TAC + MP groups, the TAC + TWG group had a lower ratio and level. In conclusion, these findings confirm that TAC + TWG has a good therapeutic effect.

Thirteen cases in the TAC + TWG group, 7 cases in the TAC group, and 5 cases in the TAC + MP group had urinary protein levels less than 0.5 g in the 10th month. According to the KDIGO clinical practice guidelines for evaluating and managing chronic kidney disease (CKD), a proteinuria level of 0.5 g/24 h is a meaningful threshold for defining the general CKD severity and high-risk populations. Therefore, the renal function prognosis was better in the TAC + TWG group than the other two groups [4, 37]. Administering TAC to treat IMN can be a financial burden in developing countries [38]. The TAC + TWG group used lower TAC doses than the other two groups (Fig. 4g), which is a strategy that can reduce treatment costs.

In 2015, Liu S et al. reported that TWG combined with corticosteroids had good efficacy for treating IMN; however, the treatment regimen had corticosteroid-related side effects [17].

Common adverse events of *Tripterygium* include stomach pain, bone marrow suppression, liver and kidney dysfunction, a decreased sperm count, and amenorrhea [39, 40]. This study investigated, for the first time, the adverse events in the three groups (Table 4). In the TAC + TWG group, only 1 case of irregular menstruation occurred. The TAC + MP group showed the highest number of newly diagnosed cases of glucose intolerance. Because TAC and MP both have an adverse effect by increasing blood glucose [30, 41], the two drugs may have additive effects on blood glucose levels. Additionally, the TAC + MP group had cases with infection and osteoporosis, which could be closely related to corticosteroid therapy [41]. However, the TAC + TWG regimen rarely led to these adverse events.

Our study had several limitations. This was a retrospective observational study, and the clinicians decided which treatment to use based on their experience. The study’s follow-up was short, and the sample size was small; thus, we could not avoid retrospective bias. However, the baseline characteristics did not significantly differ between the three groups, except in the concentrations of anti-PLA2R antibodies. Selective bias may have been too small to affect comparing the three treatment effects; therefore, we recommend conducting RCT studies to assess the efficacy and safety of TAC combined with TWG in treating IMN.

## Conclusion

TAC combined with TWG for IMN has been confirmed to result in a higher remission rate and lower relapse rate, but we recommend conducting a large-scale RCT to assess the efficacy and safety of TAC combined with TWG.

## Abbreviations

ALT: alanine aminotransferase
AST: aspartate aminotransferase
BMI: body mass index
BP: blood pressure
BUN: blood urea nitrogen
eGFR: estimated glomerular filtration rate
Hb: hemoglobin
HDL: high-density lipoprotein
LDL: low-density lipoprotein
MP: methylprednisolone
PLT: platelet count
SCr: serum creatinine
TAC: tacrolimus
Tch: total cholesterol
TG: triglyceride
TWG: tripterygium wilfordii multiglycosides
UA: uric acid
WBC: white blood cell count
